# Microstructure and Electrical Conductivity of Cement Paste Reinforced with Different Types of Carbon Nanotubes

**DOI:** 10.3390/ma15227976

**Published:** 2022-11-11

**Authors:** Alicia Páez-Pavón, Andrea García-Junceda, Andrea Galán-Salazar, Rosario G. Merodio-Perea, José Sánchez del Río, Isabel Lado-Touriño

**Affiliations:** 1 School of Architecture, Engineering and Design, Universidad Europea de Madrid, C/Tajo s/n, 28670 Villaviciosa de Odón, Spain; 2 Imdea Materials Institute, C/ Eric Kandel 2, Getafe, 28906 Madrid, Spain; 3 Department of Electrical, Electronical and Automatic Control Engineering and Applied Physics, Escuela Técnica Superior de Ingeniería y Diseño Industrial, Universidad Politécnica de Madrid, Rda. de Valencia 3, 28012 Madrid, Spain

**Keywords:** reinforced cement, multi-walled carbon nanotubes, single-walled carbon nanotubes

## Abstract

Over the last few years, the addition of small amounts of carbon nanotubes (CNTs) to construction materials has become of great interest, since it enhances some of the mechanical, electrical and thermal properties of the cement. In this sense, single-walled and multi-walled carbon nanotubes (SWCNTs and MWCNTs, respectively) can be incorporated into cement to achieve the above-mentioned improved features. Thus, the current study presents the results of the addition of SWCNTs and MWCNTs on the microstructure and the physical properties of the cement paste. Density was measured through He pycnometry and the mass change was studied by thermogravimetric analysis (TGA). The microstructure and the phases were analyzed using scanning electron microscopy (SEM) and X-ray diffraction (XRD). Finally, the electrical conductivity for different CNT concentrations was measured, and an exponential increase of the conductivity with concentration was observed. This last result opens the possibility for these materials to be used in a high variety of fields, such as space intelligent systems with novel electrical and electronic applications.

## 1. Introduction

In the construction field, concrete is one of the most frequently used materials worldwide. However, due to the emergence of porosity and cracks, the durability and performance of the cement decreases. This situation has been overcome with the introduction of nanomaterials within the cement paste structure, making it possible to produce more durable and crack-free cementitious materials [1,2,3]. In this sense, CNTs show excellent mechanical properties and chemical stability, promoting the enhancement of concrete performance. The incorporation of small concentrations of these nanostructures is causing a revolution in the construction industry since it is possible to increase both durability and mechanical performance together with electrical and thermal properties. This positive effect is making it possible to go beyond conventional construction applications due to the higher insulating sound capacity and higher thermal and electrical conductivities. This new family of cements would make it possible to manufacture sensors to monitor the internal behavior of structures and to control corrosion of metallic parts in reinforced concrete. It could also be used for cathodic protection, electromagnetic shielding or protection for electric systems. When a certain concentration of CNTs is achieved in the cement matrix, it easily forms conductive networks within the material, making it possible to employ them in anti-corrosion applications or heating mechanisms [4,5,6,7,8].

Several studies devoted to the incorporation of CNTs in concrete and cement to improve their structural and electrical properties have recently been carried out. Lazaros et al. [9] introduced semiconductor n- and p- MWCNTs to concrete in order to improve its thermoelectrical properties and to increase its energy storage capacity. Shao et al. [10] could enhance the dispersion ability of CNTs by using dispersants and, as a result, electrical conductivity was improved through the formation of electrical networks in the cement-based composite at mature stage. Elena Cerro et al. [11] researched the effects on the strength and electrical properties when adding different concentrations of MWCNTs to cement mortar. An increase of 10% in conductivity was observed in mortars with 0.01 and 0.015 wt.% MWCNT loading after 28- and 90-days curing, respectively. However, up to date, in the authors knowledge, there are no studies mixing different weight concentrations of MWCNTs with cement and reporting the average electrical conductivity for each of the cement-based samples measured at different directions (transverse and longitudinal) of the current flow. This gives a more general idea of the average electrical conductivity present in the cement-based bulk material, eliminating possible spatial fluctuations of the MWCNTs concentration inside the material volume. In this work, the average electrical conductivity results measured at different directions is determined for samples with 0.01, 0.02, 0.05 and 0.10 wt.% MWCNTs after 1 month of curing. In addition, conductivity measurements of samples with 0.02 wt.% SWCNTs incorporated to the cement matrix were performed. An exponential growth in the electrical conductivity was observed when the MWCNT weight concentration was increased. The electrical conductivity of the samples with 0.02 wt.% of SWCNTs resulted in a very similar value to the pure cement. Its morphology and shorter length than MWCNTs resulted in a less effective electrical conductivity.

The addition of other types of carbon-based nanostructures to the cement also modifies its properties, but with different effects. Wang et al. [12] observed that small additions of up to 0.3 wt.% of carbon nanofibers (CNF) have a moderate effect on the electrical resistivity of the cement, although it decreases considerably above that content. Konsta-Gdoutos et al. [13] observed that low contents of CNF in the cement, below 0.1 wt.%, provide higher values of electrical resistivity than the same content of CNTs, due to the worse dispersion in the cement paste and polarization effect.

The analysis of water absorption and the final microstructure of cement reinforced with carbon nanotubes has been studied by some authors [3,14]. In the present work, water absorption and observation of the microstructure of the materials during the setting process have been carried out, comparing them with the unreinforced material, in order to understand how the presence of carbon nanotubes affects the stages of the setting process.

Within this framework, this work proposes to analyze the formation of the microstructure during the hydration process and its achieved density, and the electrical properties reached in the cement paste with the incorporation of both single and multi-walled CNTs.

## 2. Materials and Methods

### 2.1. Materials

Portland cement (II/B-L 32.5R grade, supplied by Cementval S.L.) was used in this study, the chemical composition is shown in Table 1. This material acquires a high initial strength and it shows an excellent workability due to its high plasticity. These characteristics make it ideal to for mixing with other materials, such as carbon nanotubes. In addition, given its good resistance, it is indicated for applications where good mechanical properties are required. On the other hand, the characteristics of the CNTs used in this work are shown in Table 2.

### 2.2. Methods

#### 2.2.1. Samples Preparation

The CNTs were firstly ultra-sonicated in water in a horn sonicator (Sonoplus HD 2070, Bandelin, Berlin, Germany) for 1 h and then added to the cement powder in a weight ratio 2:1 cement–water. Cement paste was reinforced with different content of CNTs according to UNE EN 196-1:2005 [15]. A final high speed (18,000 rpm) mixing step was implemented. The fresh cement paste was casted into cylindrical molds of 2 cm height and 1 cm diameter. Plain cement paste was prepared as a control sample. The optimization of the mixing parameters of carbon nanotubes in the cement paste have been selected based on previous studies [16,17].

#### 2.2.2. Microstructure, Hydration and Density

The microstructures of the samples were observed by SEM at 7 and 28 days after their preparation. Thermogravimetric analysis (TGA) was performed in order to observe the mass change, from room temperature to 980 °C, at a heating rate of 10 °C min^−1^, under a nitrogen atmosphere. Fully setting specimens (above 30 days) were characterized by measuring the density through He pycnometry. Moreover, diffraction patterns of the samples were obtained using an X-ray diffractometer (Siemens D5000, Munich, Germany), using Cu Kα radiation and [15°–120°] 2θ range.

#### 2.2.3. Electrical Conductivity

**Figure 1:**
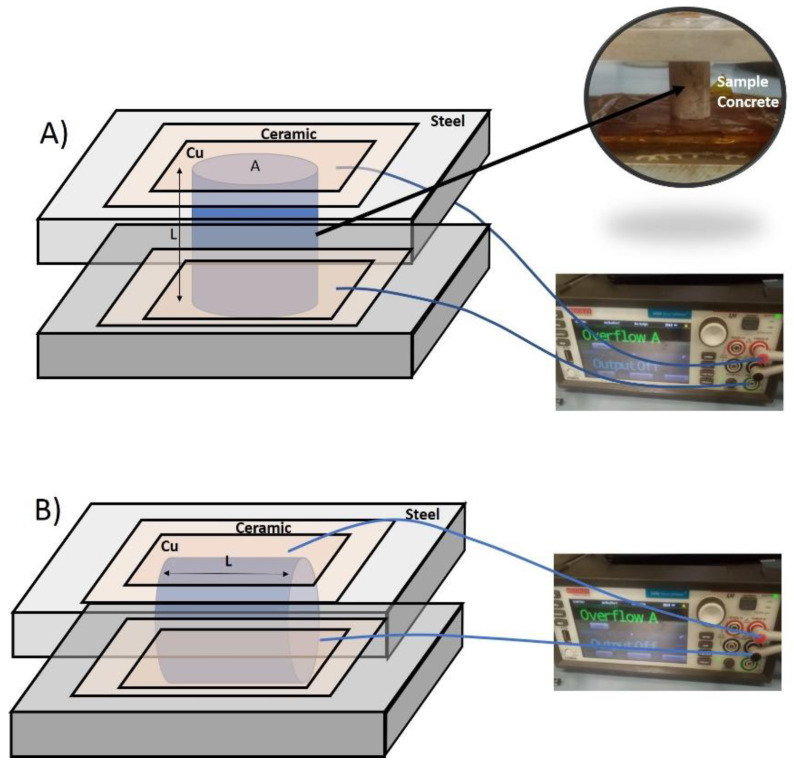
Experimental set-up to measure the electrical conductivity in (**A**) transverse direction, (**B**) longitudinal direction.

Electrical conductivity measurements were carried out with a Keithley 2450 source meter. Cement based samples had 0.00, 0.02, 0.05 and 0.1 wt.% of MWCNTs distributed in the cement structure. In addition, one of the samples had 0.02% wt.% of SWCNTs. These samples, which were cut in cylinders of 1.5 cm height and circular bases of 1 cm of diameter, were stacked between two squared steel plates of area 12 × 12 cm^2^ and 1 cm thickness, in horizontal and vertical positions. In one of the sides of the steel plates, a ceramic layer of 5 × 5 cm^2^ and 5 mm thick was fixed, and a copper layer of 1 mm was glued to this ceramic layer. Two copper-wire electrodes were attached between the plates and the ceramic layers, and were connected to the inputs of the Keithley 2450 source meter. With this configuration (see Figure 1), both sample sides were in contact with the electrodes. V–I measurements were performed with the Keithley Kickstart software. Thus, the voltage and the electrical current applied to the samples were programmed to measure in the range 10 mV–25 V and 5 nA–30 mA, respectively. Afterwards, the slopes obtained from the linear fits corresponding to the plots V–I were determined in order to obtain the resistance (R) values, either for horizontal or vertical positions. Resistivity was calculated according to these pair of resistance values and the samples geometry. When considering horizontal positions (cylinder lying on the metal plate with only a line of contact points touching the plate surface), resistivity was calculated by using the equation $\rho =\frac{R}{l}$, where R is the resistance and l corresponds to the sample thickness. In the case of the vertical ones, the used equation is $\rho =R\cdot \frac{l}{A}$, A being the transversal section of the sample perpendicular to the current flow. Once both types of resistances were calculated, their average was estimated in the samples under study in order to obtain the final conductivity, σ, by using the expression $\sigma =\frac{1}{\rho }$.

## 3. Results and Discussion

### 3.1. Microstructure, Hydration and Density

**Figure 2:**
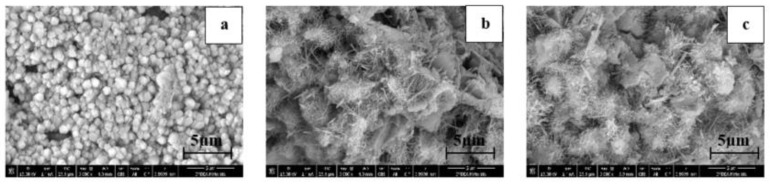
SEM micrographs 7 days after the preparation of (**a**) control sample, (**b**) 0.02 wt.% MWCNTs reinforced sample and (**c**) 0.02 wt.% SWCNTs reinforced sample.

Figure 2 shows the microstructure of the samples 7 days after their preparation. The reinforced specimens present a more developed microstructure than the control sample, in which hydration products are formed. For the unreinforced sample, the setting process is less advanced. Therefore, the obtained microstructures show a morphology consistent with the literature. As reported by Markar et al. [18], the addition of 0.02 wt.% CNTs to the cement paste directly affects the early hydration process, causing higher hydration rates, since the nanotubes act as nucleation sites for hydration products. Regarding MWCNTs, some authors have shown that there is a significant shift in the calorimetric curves, which leads to the acceleration of the hydration process, compared to the plain cement [19,20]. As for SWCNTs [18,21], the addition of this type of nanotubes to the cement paste also boosts the hydration reaction of the tricalcium silicate (C3S), which become quickly coated with calcium silicate hydrate (CSH). Further, the heat of hydration increases, since the formation of Ca (OH)_2_ is promoted during the hydration reaction.

**Figure 3:**
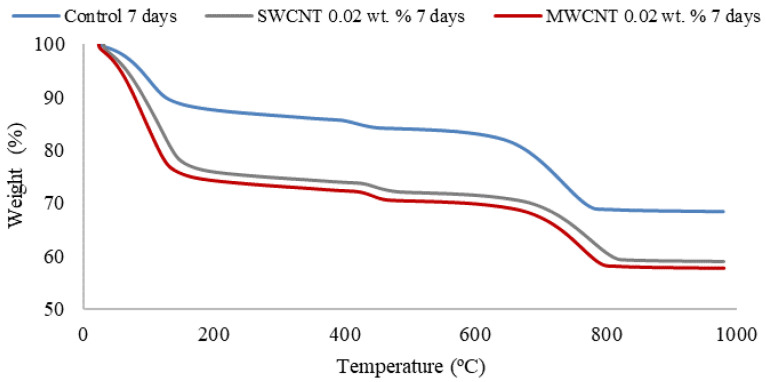
Thermogravimetric curves for the control sample, the 0.02 wt.% SWCNTs and the 0.02 wt.% MWCNTs reinforced cement samples, after 7 days of curing time.

Figure 3 shows the weight loss curves for the samples 7 days after their preparation. The first weight loss, around 100 °C, corresponds to the dehydration of CSH. This weight loss is greater for the reinforced samples, due to the presence of nanotubes leading to a greater amount of hydration products. At 450 °C, the second weight loss can be appreciated in the graphic, which is shifted to the right for reinforced samples and corresponds to the dehydroxilation of the portlandite. The final weight loss, over 650 °C, corresponds to the decarbonation of the calcium carbonate. Again, the curve for reinforced samples is shifted to the right, since the addition of both SWCNTs and MWCNTs favors the nucleation of C3S products, which are rapidly coated with CSH. Boosting of the cement hydration leads to several benefits, including earlier surface finishing, reduction of hydraulic pressure of the parts and reduction of the curing time [22].

**Figure 4:**
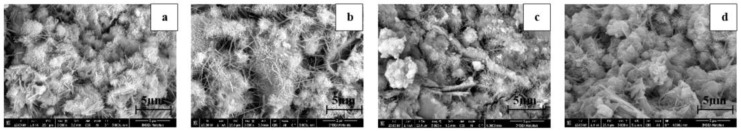
SEM micrographs 28 days after the preparation of (**a**) control sample, (**b**) 0.02 wt.% MWCNTs reinforced sample, (**c**) 0.1 wt.% MWCNTs reinforced sample and (**d**) 0.02 wt.% SWCNTs reinforced sample.

The specimens were also investigated after 28 days of curing. Figure 4 depicts the microstructure of the prepared samples, which contain different amounts of SW and MW nanotubes. As it can be seen, SEM micrographs are consistent with other studies found in the literature [23,24,25], showing a homogenous microstructure.

**Figure 5:**
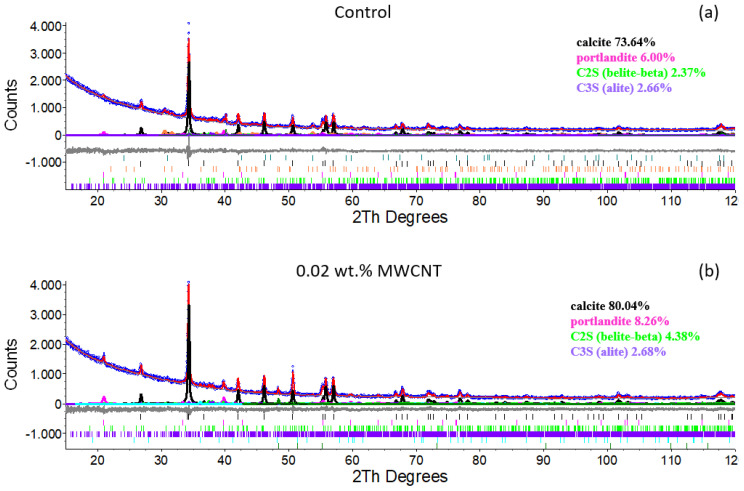

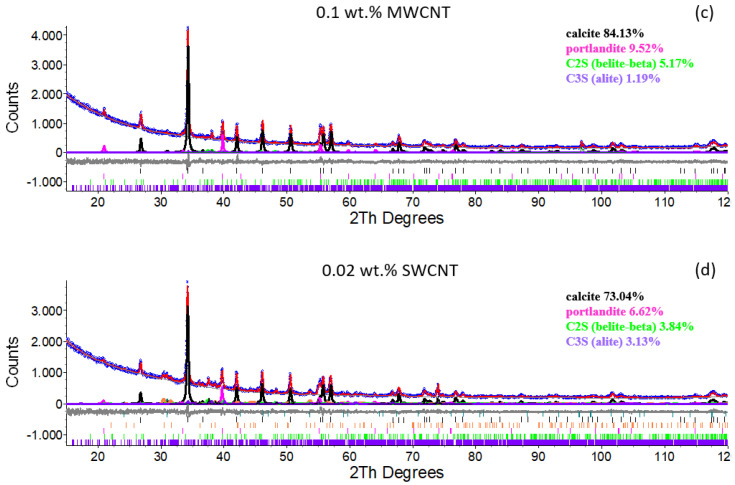
XRD spectra of (**a**) control sample, (**b**) 0.02 wt.% MWCNTs reinforced sample, (**c**) 0.1 wt.% MWCNTs reinforced sample and (**d**) 0.02 wt.% SWCNTs reinforced sample.

Moreover, the microstructure study was completed by XRD analysis of the specimens, as shown in Figure 5, in which the different hydration products are detected, being possible to observe similar patterns for all the tested conditions. Ca(OH)_2_ peak appears at 2θ = 35°, calcium carbonate at both 2θ = 21°and 2θ = 40°, while CSH is shown at 2θ = 28°. The intensity of Ca(OH)_2_ and CSH peaks are of huge relevance, since they allow to identify the degree of hydration. As is noticeable in Figure 5, the peak intensity of the CSH at 2θ = 28° increases in the presence of the CNTs, which means a positive effect of the CNTs in the hydration process of the cementitious material. The enhanced performance of the hydration reaction is a consequence of the CNTs role as nucleation sites for the hydration products and the improvement in the bonding among the mentioned products [11,26,27,28].

**Figure 6:**
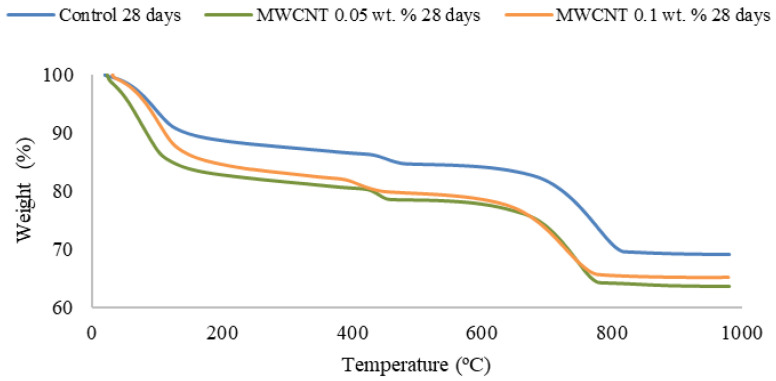
Thermogravimetric curves for the control sample, the 0.05 wt.% MWCNTs and the 0.1 wt.% MWCNTs reinforced cement samples, after 28 days of curing time.

The positive influence of the nanotubes on the hydration rate of the cementitious material is also observed in the thermogravimetric graph displayed in Figure 6, being even more significant when MWCNTs are added. This fact is related to the higher concentration of hydration products after 28 days of curing time rather than after 7 days.

The pycnometer density values for all samples are listed in Table 3. The addition of CNTs to the cement paste slightly decrease the density with regard to the control specimen, due to the larger number of pores in the CSH gel [29].

### 3.2. Electrical Conductivity

**Figure 7:**
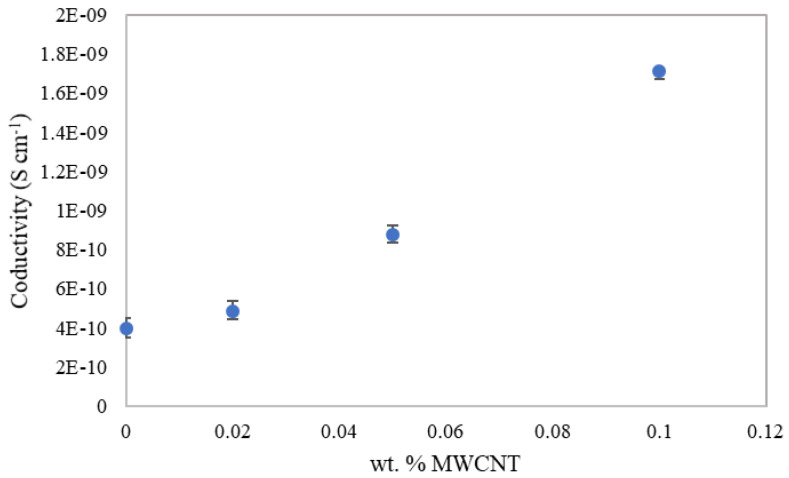
Electrical conductivity of the control sample and cementitious samples reinforced with different amounts of MWCNTs.

An exponential growth of the electrical conductivity was observed with the increase of the weight percentage of MWCNTs in the samples at room temperature (Figure 7). All measurements performed on each of the samples were repeated three times and errors were calculated by using the standard deviation for each of these groups of three measurements. Results were obtained by calculating the average of the longitudinal and transversal conductivities measured for every MWCNT concentration sample and including their corresponding errors. Conductivity of the concrete without CNTs was 3.95 10^−10^ S cm^−1^. When adding a concentration of 0.1% by weight of MWCNTs, conductivity was 4.3 times higher than the one measured in the control sample (without any amount of MWCNT). In the current study, the relative increase in the electrical conductivity of the reinforced samples with respect to the reference was higher than that obtained in other research [6,30]. The electrical conductivity was increased because the nanotubes forming a conductive network allowed the movement of ions through it. Moreover, the higher the loading of carbon nanotubes in the cement, the higher the effectiveness of the conductive network. These results are consistent with those obtained in other investigations [31,32]. Conductivity of the 0.02 wt.% SWCNT cement-based samples was also measured, resulting in (4.0 ± 0.8) × 10^−10^ S m^−1^, which is much lower than the other ones. The morphology of the MWCNTs seems to be more effective in improving the electrical conductivity than the SWCNTs, as reported in different studies. In addition, the length of the MWCNT used in this study is greater than that of the SWCNTs (see Table 2). According to the bibliography, the increase of length of the carbon nanotubes has a positive effect on the electrical conductivity [33,34,35].

## 4. Conclusions

As a main conclusion of the current study, it has been determined that the addition of CNTs to the plain cement accelerates the formation of hydration products, clearly observable at the early curing stages within the matrix (7 days). However, small variations in the quantities of CNTs added to the cement do not significantly modify the resulting microstructure of the cementitious material after the same curing process. In the same way, the type of nanotube added to the cement (single or multi-walled) does not affect the final microstructure achieved in the material. Thus, CNTs influence the hydration reaction of the cement paste, since they positively contribute to the hydration rate of the samples, causing the acceleration in the microstructure formation. Subsequent ideas that can be highlighted are listed as follows:

- The specimens reinforced with CNTs are less dense than the plain cement samples due to the increased formation of pores in the CSH gel fraction.
- Electrical conductivity was measured for different cement-based samples including plain cement, 0.02%, 0.05% and 0.1 wt.% of MWCTNs after 30 days of curing. Results show an exponential growth with the amount of MWCNTs due to the formation of a conductive network whose effectiveness improves with the loading of nanotubes. In addition, electrical conductivity of cement-based samples with 0.02 wt.% of SWCNTs was also measured resulting in a very similar value to the pure cement. The morphology of MWCNTs is more effective improving the electrical conductivity than the morphology of SWCNTs.

## Figures and Tables

**Table 1:** Chemical composition of Portland II/B-L 32.5R cement (provided by the supplier).

Component	Amount (wt.%)
SiO_2_	12.59
Al_2_O_3_	3.10
Fe_2_O_3_	1.94
CaO	59.04
TiO_2_	0.19
MnO	0.04
P_2_O_5_	0.10
SrO	0.04
MgO	1.82
K_2_O	0.58
SO_3_	2.48

**Table 2:** Carbon nanotube characteristics.

Type of Carbon Nanotube	Average Diameter (nm)	Average Length (µm)	Purity (%)	Provider
Multi-walled (MWCNT)	12	10	>98	Sigma Aldrich
Single-walled (SWCNT)	0.78	1	>95	Sigma Aldrich

**Table 3:** Density results of control sample and CNTs reinforced cement samples.

	Control	MWCNT 0.02 wt.%	MWCNT 0.1 wt.%	SWCNT 0.02 wt.%
Pycnometer density (g cm^−3^)	2.58	2.37	2.47	2.38

## References

[1] Peyvandi A., Soroushian P., Abdol N., Balachandra A.M. (2013). Surface-Modified Graphite Nanomaterials for Improved Reinforcement Efficiency in Cementitious Paste. Carbon.

[2] Alrekabi S., Cundy A., Lampropoulos A., Savina I. (2016). Experimental Investigation on the Effect of Ultrasonication on Dispersion and Mechanical Performance of Multi-Wall Carbon Nanotube-Cement Mortar Composites. Int. J. Civ. Environ. Struct. Constr. Archit. Eng..

[3] Xu S., Liu J., Li Q. (2015). Mechanical Properties and Microstructure of Multi-Walled Carbon Nanotube-Reinforced Cement Paste. Constr. Build. Mater..

[4] Tian X., Hu H. (2012). Test and Study on Electrical Property of Conductive Concrete. Procedia Earth Planet. Sci..

[5] Yunchuan Z., Liang B., Shengyuan Y., Guting C. (2012). Simulation Analysis of Mass Concrete Temperature Field. Procedia Earth Planet. Sci..

[6] Konsta-Gdoutos M.S., Batis G., Danoglidis P.A., Zacharopoulou A.K., Zacharopoulou E.K., Falara M.G., Shah S.P. (2017). Effect of CNT and CNF Loading and Count on the Corrosion Resistance, Conductivity and Mechanical Properties of Nanomodified OPC Mortars. Constr. Build. Mater..

[7] Shang Y., Zhang D., Yang C., Liu Y., Liu Y. (2015). Effect of Graphene Oxide on the Rheological Properties of Cement Pastes. Constr. Build. Mater..

[8] Rashad A.M. (2017). Effect of Carbon Nanotubes (CNTs) on the Properties of Traditional Cementitious Materials. Constr. Build. Mater..

[9] Tzounis L., Liebscher M., Fuge R., Leonhardt A., Mechtcherine V. (2019). P- and n-Type Thermoelectric Cement Composites with CVD Grown p- and n-Doped Carbon Nanotubes: Demonstration of a Structural Thermoelectric Generator. Energy Build..

[10] Shao H., Chen B., Li B., Tang S., Li Z. (2017). Influence of Dispersants on the Properties of CNTs Reinforced Cement-Based Materials. Constr. Build. Mater..

[11] Cerro-Prada E., Pacheco-Torres R., Varela F. (2021). Effect of Multi-Walled Carbon Nanotubes on Strength and Electrical Properties of Cement Mortar. Materials.

[12] Wang H., Gao X., Liu J., Ren M., Lu A. (2018). Multi-Functional Properties of Carbon Nanofiber Reinforced Reactive Powder Concrete. Constr. Build. Mater..

[13] Konsta-Gdoutos M.S., Aza C.A. (2014). Self Sensing Carbon Nanotube (CNT) and Nanofiber (CNF) Cementitious Composites for Real Time Damage Assessment in Smart Structures. Cem. Concr. Compos..

[14] Chaipanich A., Nochaiya T., Wongkeo W., Torkittikul P. (2010). Compressive Strength and Microstructure of Carbon Nanotubes–Fly Ash Cement Composites. Mater. Sci. Eng. A.

[15] (2005). Methods of Testing Cement. Part 1: Determination of Strength.

[16] Talayero C., Aït-Salem O., Gallego P., Páez-Pavón A., Merodio-Perea R.G., Lado-Touriño I. (2021). Computational Prediction and Experimental Values of Mechanical Properties of Carbon Nanotube Reinforced Cement. Nanomaterials.

[17] Páez Pavón A., Touriño L., Isabel M., Asenjo Álvarez F., Caballero Montes J.A., Cerpa Naranjo A., Galindo Muñoz A., Alanbari Ali Hassan M.H., García Junceda A. Effect of Single-Walled Carbon Nanotubes on the Physical Properties of Cement Paste. Proceedings of the ISER 174th International Conference.

[18] Makar J., Margeson J., Luh J. Carbon Nanotube/Cement Composites-Early Results and Potential Applications. Proceedings of the 3rd International Conference on Construction Materials: Performance, Innovations and Structural Implications.

[19] Stynoski P., Mondal P., Marsh C. (2015). Effects of Silica Additives on Fracture Properties of Carbon Nanotube and Carbon Fiber Reinforced Portland Cement Mortar. Cem. Concr. Compos..

[20] Cui H., Yang S., Memon S.A. (2015). Development of Carbon Nanotube Modified Cement Paste with Microencapsulated Phase-Change Material for Structural–Functional Integrated Application. Int. J. Mol. Sci..

[21] Makar J.M., Chan G.W. (2009). Growth of Cement Hydration Products on Single-Walled Carbon Nanotubes. J. Am. Ceram. Soc..

[22] Payá J., Monzó J., Borrachero M.V., Velázquez S. (2003). Evaluation of the Pozzolanic Activity of Fluid Catalytic Cracking Catalyst Residue (FC3R). Thermogravimetric Analysis Studies on FC3R-Portland Cement Pastes. Cem. Concr. Res..

[23] Szeląg M. (2017). Mechano-Physical Properties and Microstructure of Carbon Nanotube Reinforced Cement Paste after Thermal Load. Nanomaterials.

[24] Adhikary S.K., Rudžionis Ž., Rajapriya R. (2020). The Effect of Carbon Nanotubes on the Flowability, Mechanical, Microstructural and Durability Properties of Cementitious Composite: An Overview. Sustainability.

[25] Parveen S., Rana S., Fangueiro R., Paiva M.C. (2015). Microstructure and Mechanical Properties of Carbon Nanotube Reinforced Cementitious Composites Developed Using a Novel Dispersion Technique. Cem. Concr. Res..

[26] Tragazikis I.K., Kordatou T.Z., Exarchos D.A., Dalla P.T., Matikas T.E. (2021). Monitoring the Hydration Process in Carbon Nanotube Reinforced Cement-Based Composites Using Nonlinear Elastic Waves. Appl. Sci..

[27] Irshidat M.R., Al-Nuaimi N., Rabie M. (2021). Influence of Carbon Nanotubes on Phase Composition, Thermal and Post-Heating Behavior of Cementitious Composites. Molecules.

[28] Fehervari A., MacLeod A.J.N., Garcez E.O., Aldridge L., Gates W.P., Yang Y., Collins F. (2020). On the Mechanisms for Improved Strengths of Carbon Nanofiber-Enriched Mortars. Cem. Concr. Res..

[29] Chen J., Akono A.-T. (2020). Influence of Multi-Walled Carbon Nanotubes on the Hydration Products of Ordinary Portland Cement Paste. Cem. Concr. Res..

[30] Jang S.-H., Kawashima S., Yin H. (2016). Influence of Carbon Nanotube Clustering on Mechanical and Electrical Properties of Cement Pastes. Materials.

[31] Yoo D.-Y., You I., Lee S.-J. (2017). Electrical Properties of Cement-Based Composites with Carbon Nanotubes, Graphene, and Graphite Nanofibers. Sensors.

[32] Dalla P.T., Dassios K.G., Tragazikis I.K., Exarchos D.A., Matikas T.E. (2016). Carbon Nanotubes and Nanofibers as Strain and Damage Sensors for Smart Cement. Mater. Today Commun..

[33] Jakubinek M.B., Johnson M.B., White M.A., Jayasinghe C., Li G., Cho W., Schulz M.J., Shanov V. (2012). Thermal and Electrical Conductivity of Array-Spun Multi-Walled Carbon Nanotube Yarns. Carbon.

[34] Park J.G., Cheng Q., Lu J., Bao J., Li S., Tian Y., Liang Z., Zhang C., Wang B. (2012). Thermal Conductivity of MWCNT/Epoxy Composites: The Effects of Length, Alignment and Functionalization. Carbon.

[35] Garcia-Macias E., D’Alessandro A., Castro-Triguero R., Pérez-Mira D., Ubertini F. (2017). Micromechanics Modeling of the Electrical Conductivity of Carbon Nanotube Cement-Matrix Composites. Compos. Part B Eng..

